# Development and Performance of a Web-Based Tool to Adjust Urine Toxicology Testing Frequency: Retrospective Study

**DOI:** 10.2196/16069

**Published:** 2020-04-22

**Authors:** Kenneth B Chapman, Martijn M Pas, Diana Abrar, Wesley Day, Kris C Vissers, Noud van Helmond

**Affiliations:** 1 Department of Anesthesiology New York University Langone Medical Center New York, NY United States; 2 The Spine & Pain Institute of New York New York, NY United States; 3 Radboud University Medical College Nijmegen Netherlands; 4 Department of Anesthesiology, Pain and Palliative Medicine Radboud University Medical Center Nijmegen Netherlands; 5 Department of Anesthesiology Cooper Medical School of Rowan University Cooper University Health Care Camden, NJ United States

**Keywords:** Urine drug testing, Opioid therapy, Chronic noncancer pain

## Abstract

**Background:**

Several pain management guidelines recommend regular urine drug testing (UDT) in patients who are being treated with chronic opioid analgesic therapy (COAT) to monitor compliance and improve safety. Guidelines also recommend more frequent testing in patients who are at high risk of adverse events related to COAT; however, there is no consensus on how to identify high-risk patients or on the testing frequency that should be used. Using previously described clinical risk factors for UDT results that are inconsistent with the prescribed COAT, we developed a web-based tool to adjust drug testing frequency in patients treated with COAT.

**Objective:**

The objective of this study was to evaluate a risk stratification tool, the UDT Randomizer, to adjust UDT frequency in patients treated with COAT.

**Methods:**

Patients were stratified using an algorithm based on readily available clinical risk factors into categories of presumed low, moderate, high, and high+ risk of presenting with UDT results inconsistent with the prescribed COAT. The algorithm was integrated in a website to facilitate adoption across practice sites. To test the performance of this algorithm, we performed a retrospective analysis of patients treated with COAT between June 2016 and June 2017. The primary outcome was compliance with the prescribed COAT as defined by UDT results consistent with the prescribed COAT.

**Results:**

979 drug tests (867 UDT, 88.6%; 112 oral fluid testing, 11.4%) were performed in 320 patients. An inconsistent drug test result was registered in 76/979 tests (7.8%). The incidences of inconsistent test results across the risk tool categories were 7/160 (4.4%) in the low risk category, 32/349 (9.2%) in the moderate risk category, 28/338 (8.3%) in the high risk category, and 9/132 (6.8%) in the high+ risk category. Generalized estimating equation analysis demonstrated that the moderate risk (odds ratio (OR) 2.1, 95% CI 0.9-5.0; *P*=.10), high risk (OR 2.0, 95% CI 0.8-5.0; *P*=.14), and high risk+ (OR 2.0, 95% CI 0.7-5.6; *P*=.20) categories were associated with a nonsignificantly increased risk of inconsistency vs the low risk category.

**Conclusions:**

The developed tool stratified patients during individual visits into risk categories of presenting with drug testing results inconsistent with the prescribed COAT; the higher risk categories showed nonsignificantly higher risk compared to the low risk category. Further development of the tool with additional risk factors in a larger cohort may further clarify and enhance its performance.

## Introduction

Despite a decline in opioid prescriptions since the height of the opioid crisis in the United States, the use of opioids for the treatment of chronic pain continues to be common, particularly among primary care physicians [1]. Chronic opioid analgesic treatment (COAT) may be associated with the development of opioid use disorders in a subset of patients [2]. To improve the safety of COAT, guidelines recommend a reduction in opioid dosage for patients prescribed high-dose COAT and monitoring of compliance with the prescribed COAT regimen [3-8].

Urine drug testing (UDT) has been suggested by several guidelines as a method to observe compliance with the prescribed therapy in patients treated with COAT [3-8]. Guidelines state that UDT should be performed at the initiation of opioid treatment [7], at least once a year for patients prescribed COAT [7], and more often for patients at higher risk of adverse consequences from COAT [6]. However, identification of high-risk patients with currently available tools may not be reliable [7]. In the absence of effective tools to identify high-risk patients, some pain physicians have advocated requiring UDT of patients every visit to increase safety through early detection of inconsistent results [9]. As a result, insurance companies have noticed a sharp increase in UDT expenditures [10] and have demanded that physicians justify performing UDT in individual patients to reduce costs [11].

Several readily available treatment-related factors are known to be associated with an increased risk of UDT results that are inconsistent with the prescribed COAT. These factors include younger age [12,13], concomitant use of a benzodiazepine [14], a history of UDT results that are inconsistent with the prescribed COAT [15], and a higher prescribed daily morphine equivalent dose [13]. We created a web-based clinical tool that uses these factors to adjust the frequency of UDT administered in a chronic noncancer pain population. The aim of this retrospective study was to validate our stratification algorithm by comparing the risk allocation of the tool and the results of drug testing over the course of 12 months.

## Methods

### Inclusion Criteria

This study was conducted in a private interventional pain management institute with 7 specialists across 4 different locations in the New York City area. We retrospectively identified patients without cancer who had chronic pain that was treated with COAT by reviewing charts between June 1, 2016 and July 1, 2016. Visits from the 12 months following the initial visit in June 2016 were reviewed for UDT results and for their consistency with the prescribed opioid therapy. The UDT Randomizer risk categories associated with each UDT result were also obtained. The UDT Randomizer risk stratification tool was implemented as part of the standard of clinical care at the institute in March 2016 and had thus been part of normal practice for some time prior to the inclusion date. Inclusion criteria for the study were age ≥18 years and treatment with opioids (extended release or immediate release) for more than 12 consecutive weeks at the start of the retrospective inclusion period. We allowed for a gap period of up to 4 weeks in opioid treatment. The underlying cause of chronic pain was retrieved from each patient’s medical record, and patients with pain due to cancer were excluded. The Staten Island University Institutional Review Board approved this study (study number: 18-0906-SIUHN) and waived the requirement to obtain informed consent for this retrospective study.

### UDT Risk Stratification and Testing Frequency With the UDT Randomizer Tool

**Figure 1:**
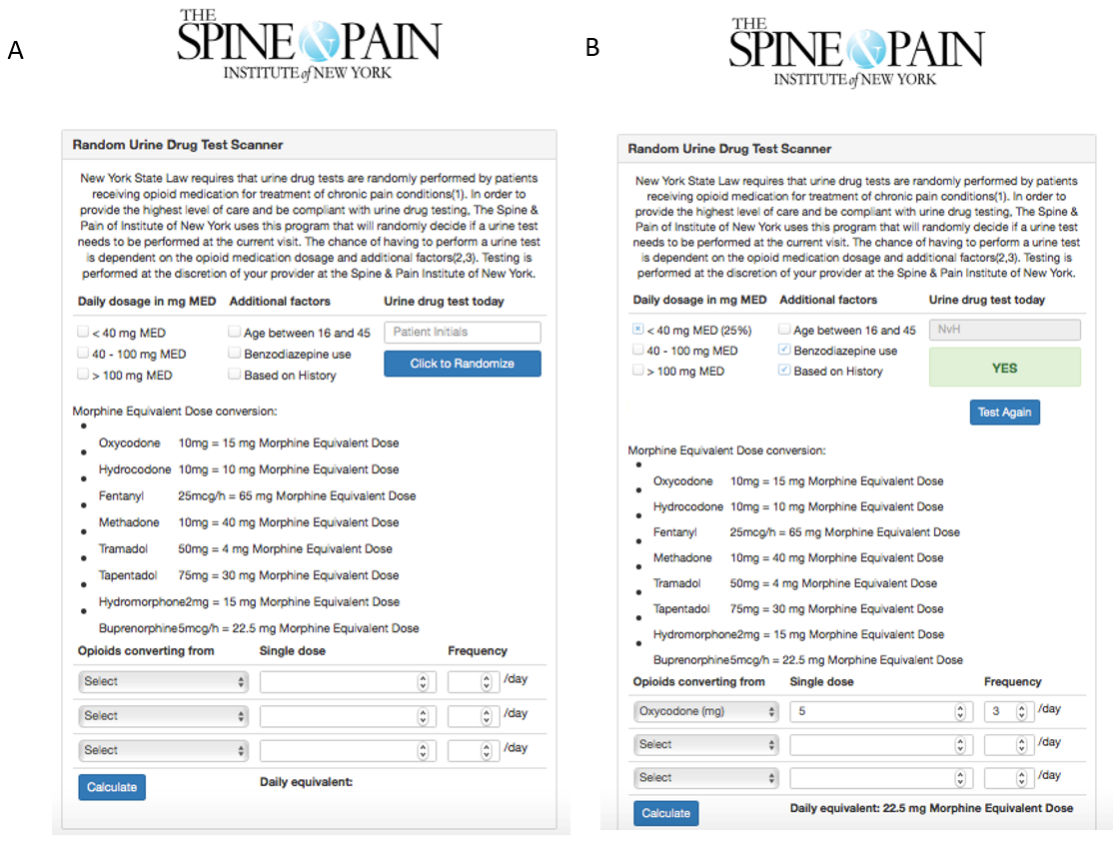
Screenshots of the UDT Randomizer tool prior to the selection of risk factors (A) and after the selection of risk factors (B). The recommendation to perform testing is “Yes” in this case.

**Figure 2:**
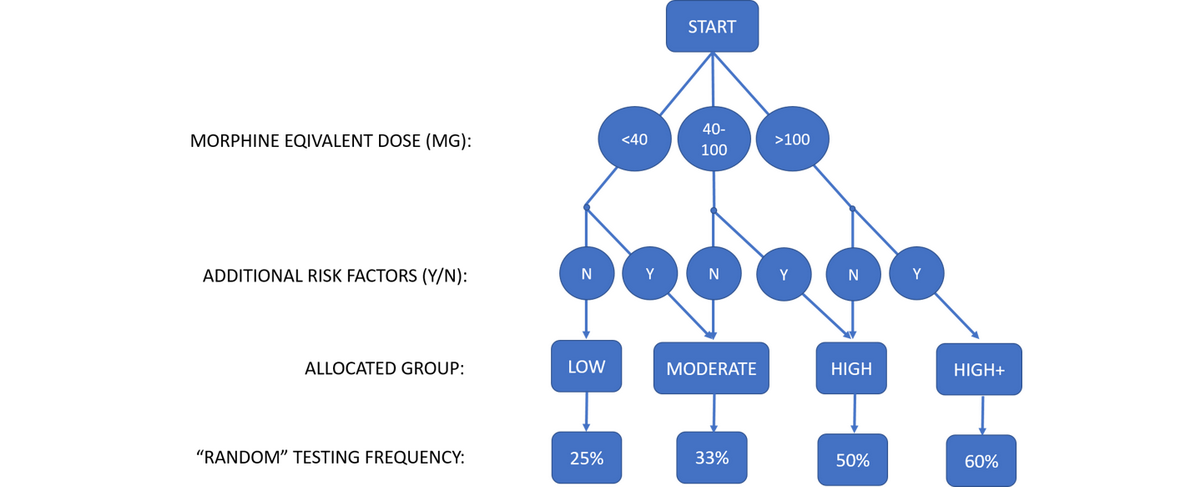
Risk category allocation and the corresponding pre-set chance that the UDT Randomizer tool will request UDT during a patient visit.

The developed stratification tool is depicted in Figure 1. Patients were assigned to a presumed risk group (low, moderate, high, or high+) based on established risk factors for UDT results inconsistent with the prescribed COAT. Patients with a history of drug testing inconsistent with the prescribed COAT are flagged in our electronic medical records, and this flag remains for the duration of treatment in our practice. Drug testing results inconsistent with the prescribed COAT may serve as an early warning of adverse outcomes of COAT [9]; therefore, we focused on developing a tool to effectively detect results inconsistent with the prescribed COAT. The risk allocation was initially based on the daily morphine equivalent dose prescribed (<40, 40-100, or >100 milligrams). The web tool incorporates a morphine equivalent dose calculator to facilitate this step. This calculator is based on a previously developed calculator [16] that was based on American Pain Society guidelines [17] and on several reviews regarding equianalgesic dosing [18-20]. When 1 or more of the additional risk factors are present (age <45 years, concomitant benzodiazepine use, or a history of drug testing results inconsistent with the prescribed COAT), the patient is escalated by 1 risk category (Figure 2).

Patients allocated to the low, moderate, high, and high+ risk categories are randomly requested to undergo UDT at frequencies of 25%, 33%, 50%, and 60%, respectively. It is important to stress that the chance of being requested to participate in UDT is thus not random but is rather random with a certain pre-set probability. We arrived at the testing frequencies through evaluation of the Washington State Agency Medical Directors’ Group Interagency Guideline and American Academy of Pain Medicine recommendations on frequency of testing [6,21]. We estimated that we would be able to achieve the recommended testing frequencies by choosing these set frequencies for the UDT Randomizer.

### Primary Outcome

The primary outcome was compliance with the prescribed opioid therapy. This was assessed by the drug test results and their consistency with the prescribed opioids over the study period. A drug test result was considered to be consistent if it was positive for the prescribed opioid or its metabolites and was negative for other opioids, their metabolites, or illicit substances. A drug test result was considered to be inconsistent if it was negative for the prescribed opioid or its metabolites or if it was positive for nonprescribed opioids, their metabolites, or illicit substances. Consistent with recent Centers for Disease Control and Prevention (CDC) guidance [7], we did not take into account the results of testing for tetrahydrocannabinol (THC) when determining if a UDT was consistent or inconsistent with the prescribed therapy.

### Drug Testing

Urine toxicology testing was performed by an independent laboratory using liquid chromatography tandem mass spectrometry (Triple Quad 4500 MD, AB Sciex). If a patient was not able to provide a urine sample, oral fluid was collected for analysis. Both urine and oral fluid samples were examined for the presence of prescribed opioids, benzodiazepines, illicit drugs, and their respective metabolites. Chromatographic tests are specific and are not susceptible to cross-reactions; thus, false positive results are rare [22]. The detection window is substantially shorter for oral fluid testing vs urine testing (eg, morphine is detectable 2-5 days after use in urine vs 1-36 hours in oral fluid [23]). When a drug is within the detection windows for both UDT and oral fluid testing, the detection rates are believed to be similar [24].

### Data Retrieval

Patient demographics, diagnoses, prescribed medications, and drug testing results were collected retrospectively from the patients’ medical records. Pain diagnoses were grouped into categories of lower back pain; cervical pain; arthritis, joint, and muscle pain; and other pain. We retrieved information from all visits in the 12-month period following the initial included visit in June 2016. Because the data analysis was conducted at the individual visit level (see the Data and Statistical Analysis section), we included data regardless of whether the patients remained in our care for the full 12-month period.

### Data and Statistical Analysis

Demographics and clinical data are presented as mean (SD) or as n (%). To assess the uptake of the UDT Randomizer tool, we analyzed how often the tool was used during the first visit for each patient in the study period. We also assessed how often the tool’s recommendation (Yes or No for UDT) was followed at that visit. Additionally, we assessed how often UDT testing was ordered without recommendation by the tool over the course of the entire study period as well as how often the UDT testing recommended by the tool was ignored by providers over the course of the entire study period. We performed generalized estimating equations (GEE) analysis with the factors “risk category” and “visit” to assess if the assigned risk category was related to the consistency of drug testing results with the prescribed COAT using all tests and risk assignments in the 12-month study period. We used GEE to account for repeated testing in the same patient. To assess our assumption that there is no association between marijuana use and drug testing results inconsistent with the prescribed COAT, we performed GEE analysis with THC status on drug testing as a factor for the consistency of drug testing results with the prescribed COAT as the outcome. The results of the analyses are presented as odds ratios (ORs) with 95% confidence intervals and the corresponding *P* values. Statistical significance was set at *P*<.05. The software package SPSS version 24 (IBM Corporation) was used for all statistical analyses.

## Results

### Study Population

The study population consisted of 320 patients, of whom 172 (53.8%) were female and 148 (46.3%) were male (Table 1). Most of the patients’ diagnoses (214/320, 66.9%) were related to spinal pain.

**Table 1:** Demographic and treatment characteristics of the patients included in the study.

Characteristic		Patients (N=320)	
Age, mean (SD)		57 (12)	
**Gender, n (%)**			
	Male		148 (46.3)
	Female		172 (53.8)
**Pain diagnosis, n (%)**			
	Lower back		214 (66.9)
	Cervical		74 (23.1)
	Arthritis, joint, and muscle		22 (7.9)
	Other^a^		10 (3.1)
Prescribed opioid dosage in morphine milligram equivalents/day, mean (SD)		70 (66)	
Concomitant use of benzodiazepines, n (%)		91 (28.4)	

^a^Patients in this category were diagnosed with abdominal pain, endometriosis, pelvic pain, fibromyalgia, phantom limb pain, or trigeminal neuralgia.

We found that the uptake of the UDT Randomizer tool was high at the first visit in the study period: it was used in 318/320 patients (99.3%), and its recommendation regarding testing was followed 314 of the 318 times it was used (99.7%). Over the course of the entire 12-month study period, the recommendation of the tool to test (“Yes”) was followed in 945/964 (98.0%) of visits. Over the 12-month period, 34 tests were performed contrary to the tool’s guidance to not perform a test.

### Primary Outcome: Drug Testing Consistency with the Prescribed COAT

A total of 979 drug tests were performed in the study population over the retrospective 12-month duration of the study. Of the performed tests, 867/979 (88.6%) were urine drug tests, whereas 112 (11.4%) were oral fluid tests. All patients provided at least 1 drug test during the follow-up period. Inconsistent drug test results were registered for 76/979 tests (7.8%) in 52/320 patients (16.3%) during this period. The incidence of inconsistent test results across the UDT Randomizer tool risk categories varied from 4.4% (low risk) to 9.2% (moderate risk), 8.3% (high risk), and 6.8% (high+ risk; Table 2).

Of the 979 drug tests, 119 (12.2%) were positive for THC, and the positive tests were obtained in 25/320 patients (7.8%). GEE analysis with the risk factors “THC” and “visit” did not demonstrate significantly higher risk of drug testing inconsistent with the prescribed COAT when a positive test for THC was also present (OR 1.3, 95% CI 0.6-3.0; *P*=.48).

### Relationships Between the Risk Tool Categories and the Consistency of UDT Results With the Prescribed COAT

GEE analysis revealed that tests in the moderate, high, and high+ risk categories were associated with a nonsignificantly higher risk of inconsistency with the prescribed COAT (Table 3).

Because the ORs appeared to be homogenous among the moderate, high, and high+ categories, we performed a secondary GEE analysis to explore the value of stratifying patients into only 2 risk categories as a potential next step in the development of the UDT Randomizer tool. We combined the previous moderate, high, and high+ categories into one high risk category. The performance of this stratification with regard to the consistency of drug testing with the prescribed opioid therapy was found to be similar to that of the individual categories in the initial 4-category system (OR of high vs low: 2.0, 95% CI 0.9-4.7; *P*=.09).

Additionally, we explored whether a lower cutoff point of 20 daily morphine milligram equivalents prescribed could improve discrimination by the UDT Randomizer tool. GEE analysis indicated that this cutoff did not perform better than the previous 2-risk category stratification (OR of high vs low: 1.4, 95% CI 0.4-4.8; *P*=.60).

**Table 2:** Consistency of drug tests with the prescribed opioid therapy in the 4 risk categories of the UDT Randomizer tool.

Result		Risk category			
		Low (n=160)	Moderate (n=349)	High (n=338)	High+ (n = 132)
**Drug test result, n (%)**					
	Consistent	153 (95.6)	317 (90.8)	310 (91.7)	123 (93.2)
	Inconsistent	7 (4.4)	32 (9.2)	28 (8.3)	9 (6.8)
**Inconsistency of result, n (%)**					
	Negative for prescribed opioid	3 (1.9)	13 (3.7)	12 (42.9)	3 (33.3)
	Positive for unprescribed opioid	3 (1.9)	15 (4.3)	12 (42.9)	6 (66.7)
	Positive for illicit drug	1 (0.6)	4 (1.1)	4 (14.3)	0 (0)

**Table 3:** Generalized estimating equations analysis of the influence of the UDT Randomizer risk category on the consistency of drug testing with the prescribed opioid therapy.

Risk category	OR^a^ (95% CI)	*P* value
Low	Reference	Reference
Moderate	2.1 (0.9-5.0)	.10
High	2.0 (0.8-5.0)	.14
High+	2.0 (0.7-5.6)	.20

^a^OR: odds ratio

## Discussion

### Principal Findings

The aim of this study was to assess a risk stratification algorithm we developed to adjust the drug testing frequency in patients being treated with COAT. The main findings are that the overall inconsistency of drug testing results with the prescribed COAT was low and that tests in the predefined moderate, high, and high+ risk categories had a nonsignificantly higher risk of being inconsistent with the prescribed COAT.

Based on available evidence, UDT has been suggested by several guidelines as a method to observe compliance with the prescribed therapy in patients treated with COAT [3-8]. However, none of these guidelines provide practical advice on the frequency of testing that should be employed. In the absence of such guidance, some pain physicians have adopted the policy of performing UDT virtually every visit to promote safety and to ensure compliance with regulations, leading to subsequent concerns of overutilization of UDT [10] and regulatory fines [25]. At the same time, a proportion of physicians undertest their patients, leading to risk that opioid-related adverse events will not be prevented [26]. Another common approach to testing is a standardized testing interval of every 3-4 months, which allows patients to prepare for upcoming UDT [15]. Appropriate patient selection for UDT would help limit overall expenses while maintaining a safe prescription environment. Prior tools that have been developed to estimate the risk of opioid abuse include the Screener and Opioid Assessment for Patients in Pain-Revised [27,28], the Current Opioid Misuse Measure [29], the Screening Instrument for Substance Abuse Potential [30], the Opioid Risk Tool [31] and the Diagnostic, Intractability, Risk, Efficacy [32] tool. These tools consist of 5-24 questions regarding behavioral factors and family history that impose a greater risk of opioid use disorders. These tools may require a significant time investment from both the patient and the pain physician and are dependent on the truthful responses of the patient. In the context of opioid use disorders, data generally show that such self-reporting is unreliable [33]. The urine toxicology tool we developed avoids self-reporting, and it incorporates only demographic and treatment-related factors that are readily available from the patient’s electronic medical record. Because the randomizer uses an algorithm based on treatment-related factors, the decision whether to perform UDT is not dependent on a direct decision made by a health care provider, which adds subjectivity to the decision process [34], and the algorithm returns randomization based solely on probability. Furthermore, taking the provider factor out of the equation may have a positive effect on the patient-physician relationship, as the physician is removed from the decision of whether UDT should be performed [7]. The tool can be utilized by any health care professional assisting the physician in the care of the patient, since all risk factors are readily available from the medical record. The presented approach avoids a routine schedule for testing (eg, every 3 or 4 months), which may be amenable to manipulation by patients who are prone to opioid misuse [7,15]. The results of this study indicate that at present, the tool cannot identify patients who are at significantly higher risk of presenting with testing results inconsistent with their prescribed COAT. There were nonsignificant differences in inconsistent UDT results between the moderate, high, and high+ categories and the low risk category. It is possible that this study is not sufficiently powered to detect differences between these groups, given the overall low incidence of inconsistent UDT results. The homogeneity of the inconsistency rates in the moderate, high, and high+ categories suggests that development of the tool should focus on combining the current moderate, high, and high+ categories while incorporating other risk factors to effectively distinguish between higher risk and lower risk patients.

The overall level of observed consistency of the UDT results with the prescribed opioid therapy was high in the present study (83.7%). This percentage is similar to the percentage reported in a recent study by Knezevic et al [15], in which 77.2% of the observed study population was found to present with consistent UDT results. In earlier studies, these rates were found to be much lower (25%-56%) [12,35,36]. These differences may be due to increased attention to compliance with COAT and UDT among physicians in more recent studies, differences in the studied populations, or differences in the definition of a “consistent result” of UDT. In our sample, 25 patients were found to test positive for THC; however, we did not consider a positive UDT result for THC to be proof of illicit drug use. In the present study, there was no association between marijuana use and UDT results inconsistent with the prescribed COAT. In the most recent CDC opioid prescription guidelines, experts noted that it may not be useful to test for THC on UDT because it is unclear if a positive test for THC should affect patient management [7]. Earlier studies reported associations between marijuana use in chronic opioid patients and present and future opioid misuse [37]. Research in twins has suggested that early-onset marijuana use is a risk factor for developing more severe and pervasive drug use disorders [38]. Currently, fewer people in the United States perceive marijuana to be harmful compared to a decade ago [39]. Medical marijuana has been introduced in 33 states (including New York and New Jersey), and 11 states allow recreational marijuana use. In states where marijuana is legalized for medical use, chronic pain is one of the approved indications [40], and most persons acquiring medical marijuana do so for pain management [41]. It has been suggested that medical marijuana legalization reduces overall opioid prescribing and high-risk opioid use [42] by providing an alternative treatment for chronic pain. It has been suggested that medical marijuana and recreational marijuana use have opposite effects on overall opioid use and opioid misuse (ie recreational marijuana increases opioid use and opioid misuse [43]), although a recent analysis of states that legalized recreational marijuana found no increases in opioid prescriptions [44].

### Strengths and Limitations

This was a retrospective study conducted at a single institution. A strength of the study was the prospective effective implementation of the intervention in the institution prior to the evaluation in this study.

Drug testing results inconsistent with prescribed COAT have been suggested to serve as an early warning of adverse outcomes of COAT [9]; therefore, we focused on developing a tool to effectively detect inconsistent results. However, the ultimate relationship between the implementation of UDT in the management of patients treated with COAT and long-term adverse events of COAT is not well established at present [7], even though its value in improving safety is assumed in several guidelines [3-8].

### Conclusion

The developed tool stratified patients during individual visits into risk categories of presenting with drug testing results inconsistent with the prescribed COAT; the higher risk categories showed nonsignificantly higher risk than the low risk category. Further development of this tool with additional risk factors in a larger cohort may further clarify and enhance its performance.

## Abbreviations

CDC: Centers for Disease Control and Prevention
COAT: chronic opioid analgesic therapy
GEE: generalized estimating equations
OR: odds ratio
THC: tetrahydrocannabinol
UDT: urine drug testing
